# Supplementary material for: Significant Sex Differences in the Efficacy of the CSF1R Inhibitor-PLX5622 on Rat Brain Microglia Elimination
Source: Pharmaceuticals (Basel). 2022 May 2;15(5):569. doi: 10.3390/ph15050569 (PMC9145577; doi:10.3390/ph15050569)
Supplement: Supplementary file 1 [file pharmaceuticals-15-00569-s001.zip › pharmaceuticals-1682130-supplementary.pdf]

Supplemental information

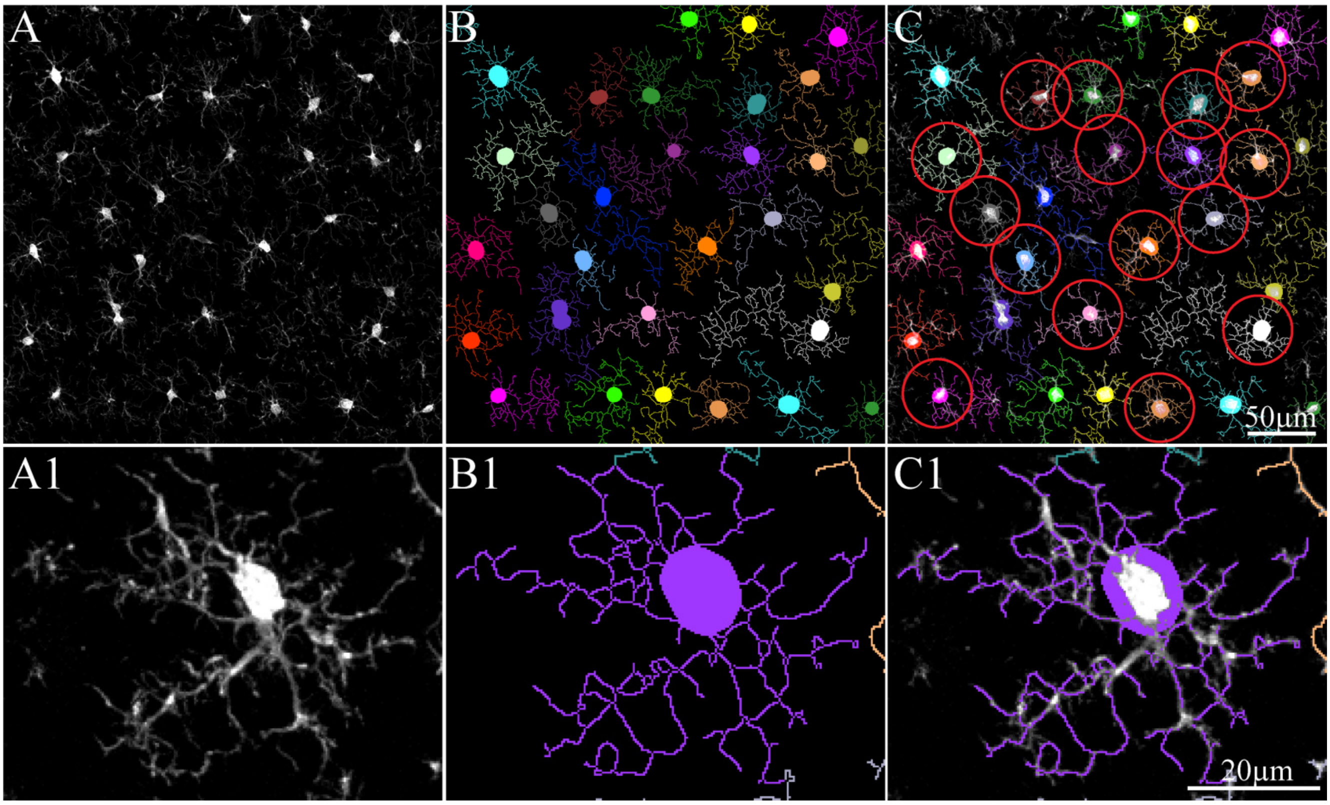

Supplementary Figure S1. Sequence of microglia morphological analysis. (A, A1) Maximal intensity projection images produced from z-stacks taken by confocal microscope. (B, B1) The cell bodies and extensions detected by HCA-vision software. (C, C1). Merge image of A/A1 and B/B1. Only the cells marked by red circle were taken for the final analysis. Cells that were cut at the edges of the image were not included in the analysis.

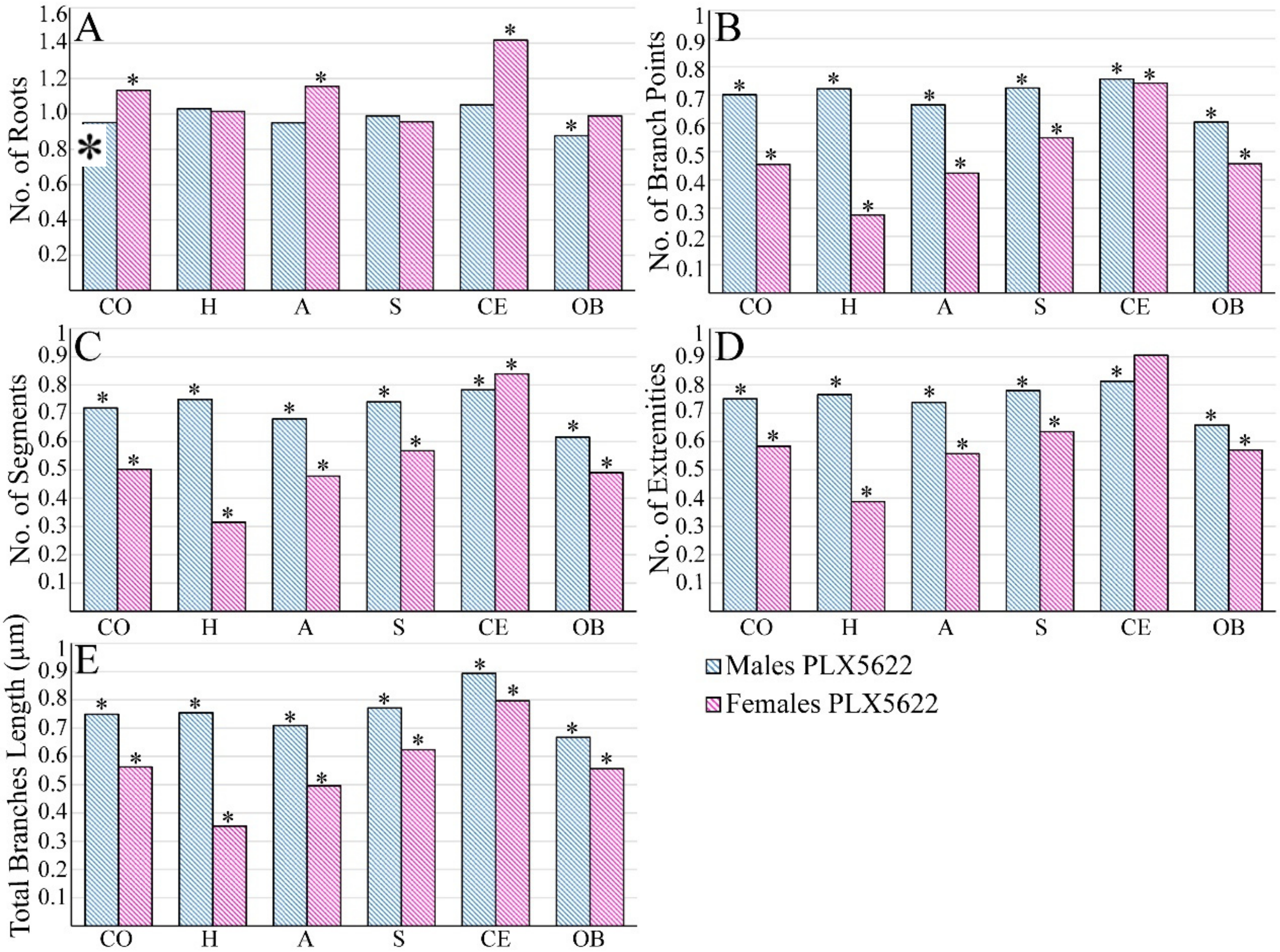

Supplementary Figure S2. Ratio values of the numbers of (A) roots/cell, (B) branching points, (C) segments, (D) extremities and (E) total branch lengths in microglia surviving 10 days of PLX5622-chow compared to the control rats in different brain regions. In females microglia surviving PLX5622 showed a reduction in all parameters and brain regions. CO-cortex, H-hippocampus, A-amygdala, S-striatum, CE-cerebellum, OB- olfactory bulb. * (Asterisk) indicate statistical significance (P≤0.01) between control and PLX5622 fed rats.

Supplementary Table S1: Density and morphology sample size, means and standard deviations of microglia in male and female rats.

Brain's Area	Group	Group size (Density)*	Density (Number of cells/mm2)	Group size (Morphology)*	Number Of Roots (R)	Number of Branch Points (B)	Number Of Segments (S)	Number Of Extremities (E)	Total Neurite Length (µm)
**Cortex**	Male	4H, 30S	252.7±29.1	20S, 247C	9.8±3.2	37.6±15.1	113.5±44.2	48.6±17.4	365±130.9
	Male PLX5622	4H, 20S	176.4±32.6	18S, 227C	9.3±3.1	26.4±12.3	81.6±35.5	36.5±13.9	273.3±102.2
	Female	4H, 28S	294.5±31.5	18S, 236C	9.8±3.1	39.4±22	118±62.5	49±22.4	370.5±167.5
	Female PLX5622	4H, 18S	28.7±11.2	96S, 217C	11.1±3.1	17.9±9.9	59.3±29.5	28.6±11.3	208.6±88.6
**Hippocampus**	Male	4H, 20S	271.2±26.6	20S, 202C	10.7±3.6	44.2±20.8	134±59.4	55.6±22.2	413.4±172.2
	Male PLX5622	4H, 20S	205.4±14.3	20S, 166C	11±3.3	31.9±16.9	100.3±50	42.6±18.5	311.8±136.4
	Female	4H, 20S	268.3±18.4	20S, 230C	11.1±3.6	57.4±26.6	173±77.5	69.1±28.8	509.7±210.7
	Female PLX5622	4H, 20S	36.2±9.4	80S, 138C	11.3±4	15.8±8.7	54.4±25.8	26.8±10.7	180.2±76.8
**Amygdala**	Male	4H, 20S	314.3±31.7	20S, 379C	10.7±3.1	39.4±18.2	119.7±52.7	49.8±19.6	378.7±149.8
	Male PLX5622	4H, 20S	227±14.1	19S, 243C	10.2±3.2	26.2±12.4	81.4±34.7	36.7±13.2	268.6±103.8
	Female	4H, 20S	281.9±25.4	19S, 324C	9.8±3	43.3±21.1	129.4±59.9	53.2±22.1	410.5±180.1
	Female PLX5622	4H, 20S	44.2±15.8	72S, 200C	11.4±3.6	18.3±10.3	61.9±31	29.6±12	203.5±88.9
**Striatum**	Male	4H, 20S	262.6±16.8	20S, 233C	10.7±3.4	45.3±23	135.9±66	55.8±24.1	442.3±191.8
	Male PLX5622	4H, 20S	186.4±30	20S, 219C	10.5±3.6	32.8±17.1	100.6±49.9	43.5±18.9	341.1±148.9
	Female	4H, 18S	247.6±18.8	20S, 176C	11.2±3.5	50.3±24.2	149.7±69.5	60.6±25.7	472.3±196.9
	Female PLX5622	4H, 19S	84±15.4	80S, 421C	10.7±3.9	27.6±14.4	85±42	38.5±16.1	294.6±122.1
**Cerebellum**	Male	4H, 19S	235±17.2	20S, 181C	7.2±2.7	21.9±10.7	66.7±31.4	31±12.3	231.9±96.9
	Male PLX5622	4H, 20S	152.6±41.6	20S, 141C	7.6±3.2	16.6±8.2	52.3±23.7	25.2±10.1	207.2±88.8
	Female	4H, 16S	228.4±25.4	20S, 196C	5.9±2.4	16.3±8.7	50.3±25.1	23.7±10.4	180±82.9
	Female PLX5622	4H, 20S	28.8±15.8	80S, 153C	8.4±3.4	12.1±6.9	42.2±21.3	21.5±9.5	143.4±63.1
**Olfactory bulb**	Male	4H, 19S	390.6±36.5	19S, 322C	8.5±3.1	29±18.9	91.3±55.4	38.4±20.3	267.7±153
	Male PLX5622	4H, 20S	374.4±38.6	20S, 268C	7.5±3.6	17.5±11.6	56.2±33.5	25.3±13.8	178.6±107.7
	Female	4H, 18S	380.8±33.8	19S, 330C	8.1±3.2	27.8±16.6	88.6±49.4	37.3±19	259±136.3
	Female PLX5622	4H, 19S	196.6.1±24.8	46S, 353C	8±3.3	12.7±7.1	43.4±21.4	21.3±8.9	144.1±68.8

* H= hemispheres, S=slices, C= cells

Supplementary Table S2: T tests of microglia density and morphology in male and female rats (P>0.01, P<0.01).

Density

Area	Female control versa Male control	Female control versa Female PLX	Male control versa Male PLX	Female PLX versa Male PLX
**Cortex**	1.28232E-06	4.93603E-32	1.47044E-10	2.7351E-16
**Hippocampus**	0.343538842	2.72361E-29	5.68626E-11	2.59566E-31
**Amygdala**	0.000509723	1.32327E-27	8.67007E-12	2.03966E-32
**Striatum**	0.00727998	2.66118E-25	2.90375E-11	2.3705E-14
**Cerebellum**	0.188266419	3.15884E-20	6.20764E-09	2.88576E-12
**Olfactory bulb**	0.199825468	8.10872E-19	0.09289157	2.38809E-18

Number Of Roots

Area	Female control versa Male control	Female control versa Female PLX	Male control versa Male PLX	Female PLX versa Male PLX
**Cortex**	0.48466279	5.03248E-06	0.047230387	1.03961E-09
**Hippocampus**	0.108314615	0.351139994	0.195996501	0.259877645
**Amygdala**	9.88841E-05	3.13381E-07	0.019802837	0.000112395
**Striatum**	0.058135284	0.062810578	0.359193931	0.299095526
**Cerebellum**	1.49724E-07	2.26186E-13	0.13686744	0.023037164
**Olfactory bulb**	0.03346394	0.353285939	9.88681E-05	0.036755492

Number of Branch Points

Area	Female control versa Male control	Female control versa Female PLX	Male control versa Male PLX	Female PLX versa Male PLX
**Cortex**	0.149955075	6.82347E-34	4.67864E-18	7.02304E-15
**Hippocampus**	7.28196E-09	1.90775E-64	5.81219E-10	1.56169E-22
**Amygdala**	0.005100329	5.955E-57	4.52848E-25	6.13575E-13
**Striatum**	0.018494101	4.6602E-25	7.73E-11	6.18083E-05
**Cerebellum**	3.58128E-08	3.77789E-07	3.44706E-07	4.63909E-07
**Olfactory bulb**	0.191980437	1.06921E-42	1.3208E-18	1.70569E-09

Number of Segment

Area	Female control versa Male control	Female control versa Female PLX	Male control versa Male PLX	Female PLX versa Male PLX
**Cortex**	0.181495104	8.32279E-32	2.79174E-17	1.15705E-12
**Hippocampus**	3.53358E-09	1.38403E-62	4.0334E-09	2.66004E-21
**Amygdala**	0.012198736	1.09764E-51	8.71499E-26	4.38264E-10
**Striatum**	0.02089021	7.84279E-25	1.65211E-10	4.50286E-05
**Cerebellum**	2.4979E-08	0.000636165	1.8258E-06	8.95111E-05
**Olfactory bulb**	0.259432673	4.17813E-43	4.19607E-20	4.29791E-08

Number Of Extremities

Area	Female control versa Male control	Female control versa Female PLX	Male control versa Male PLX	Female PLX versa Male PLX
**Cortex**	0.426569028	7.91686E-30	2.50976E-16	3.89412E-11
**Hippocampus**	3.46212E-08	9.97524E-59	1.09182E-09	2.7861E-18
**Amygdala**	0.014837858	2.19313E-46	6.16029E-22	3.09508E-09
**Striatum**	0.025884183	5.70877E-22	1.66788E-09	0.000474674
**Cerebellum**	7.68918E-10	0.01772186	2.46424E-06	0.000586579
**Olfactory bulb**	0.245116391	1.40672E-37	1.53792E-19	2.18814E-05

Total branches Length

Area	Female control versa Male control	Female control versa Female PLX	Male control versa Male PLX	Female PLX versa Male PLX
**Cortex**	0.342899156	2.53799E-32	1.03102E-16	1.94794E-12
**Hippocampus**	1.3905E-07	5.72807E-64	3.78793E-10	2.2061E-22
**Amygdala**	0.00607278	2.98931E-54	2.1957E-25	2.25042E-12
**Striatum**	0.06143642	1.29616E-23	4.02957E-10	4.16974E-05
**Cerebellum**	2.56074E-08	2.05095E-06	0.008992885	8.77326E-12
**Olfactory bulb**	0.222209133	7.67028E-37	4.71782E-16	3.02175E-06

Density

Males	Cortex	Hippocampus	Amygdala	Striatum	Cerebellum	Olfactory bulb
Cortex		0.012311669	1.39128E-08	0.067174482	0.005118193	1.9732E-15
Hippocampus	0.012311669		2.00808E-05	0.114212007	7.23684E-06	1.5833E-13
Amygdala	1.39128E-08	2.00808E-05		2.37547E-07	3.87275E-11	1.84807E-08
Striatum	0.067174482	0.114212007	2.37547E-07		5.89755E-06	1.3185E-13
Cerebellum	0.005118193	7.23684E-06	3.87275E-11	5.89755E-06		8.53606E-16
Olfactory bulb	1.9732E-15	1.5833E-13	1.84807E-08	1.3185E-13	8.53606E-16	
**Females**	**Cortex**	**Hippocampus**	**Amygdala**	**Striatum**	**Cerebellum**	**Olfactory bulb**
Cortex		0.000367897	0.065159985	5.76098E-08	1.25687E-09	1.52621E-10
Hippocampus	0.000367897		0.03061077	0.000810422	5.15008E-06	7.73132E-13
Amygdala	0.065159985	0.03061077		1.68424E-05	1.48991E-07	1.21065E-11
Striatum	5.76098E-08	0.000810422	1.68424E-05		0.008490304	1.21802E-14
Cerebellum	1.25687E-09	5.15008E-06	1.48991E-07	0.008490304		3.42404E-16
Olfactory bulb	1.52621E-10	7.73132E-13	1.21065E-11	1.21802E-14	3.42404E-16	

Number Of Roots

Males	Cortex	Hippocampus	Amygdala	Striatum	Cerebellum	Olfactory bulb
Cortex		0.002797316	0.000249575	0.001868477	2.51495E-18	2.48814E-06
Hippocampus	0.002797316		0.493718166	0.457777687	6.72197E-24	9.82722E-12
Amygdala	0.000249575	0.493718166		0.454611283	1.79418E-35	1.01936E-18
Striatum	0.001868477	0.457777687	0.454611283		2.03902E-27	1.79101E-13
Cerebellum	2.51495E-18	6.72197E-24	1.79418E-35	2.03902E-27		3.84886E-07
Olfactory bulb	2.48814E-06	9.82722E-12	1.01936E-18	1.79101E-13	3.84886E-07	
**Females**	**Cortex**	**Hippocampus**	**Amygdala**	**Striatum**	**Cerebellum**	**Olfactory bulb**
Cortex		1.16842E-05	0.451693433	1.5795E-05	4.50645E-40	2.6707E-10
Hippocampus	1.16842E-05		4.33518E-06	0.415111777	1.48557E-53	9.71391E-23
Amygdala	0.451693433	4.33518E-06		7.43571E-06	8.41635E-49	1.18479E-12
Striatum	1.5795E-05	0.415111777	7.43571E-06		1.05693E-45	2.88873E-20
Cerebellum	4.50645E-40	1.48557E-53	8.41635E-49	1.05693E-45		7.99237E-18
Olfactory bulb	2.6707E-10	9.71391E-23	1.18479E-12	2.88873E-20	7.99237E-18	

Number Branch Points

Males	Cortex	Hippocampus	Amygdala	Striatum	Cerebellum	Olfactory bulb
**Cortex**		9.12444E-05	0.091183052	1.04692E-05	1.96314E-31	1.3683E-09
**Hippocampus**	9.12444E-05		0.002854753	0.305315366	9.2749E-33	3.09348E-16
**Amygdala**	0.091183052	0.002854753		0.000476691	1.75823E-39	2.37045E-13
**Striatum**	1.04692E-05	0.305315366	0.000476691		9.398E-35	9.99579E-18
**Cerebellum**	1.96314E-31	9.2749E-33	1.75823E-39	9.398E-35		5.93133E-08
**Olfactory bulb**	1.3683E-09	3.09348E-16	2.37045E-13	9.99579E-18	5.93133E-08	

Females	Cortex	Hippocampus	Amygdala	Striatum	Cerebellum	Olfactory bulb
**Cortex**		7.07412E-15	0.018850437	2.04186E-06	3.96009E-38	1.62504E-11
**Hippocampus**	7.07412E-15		2.80527E-11	0.002388912	5.94995E-64	6.07646E-40
**Amygdala**	0.018850437	2.80527E-11		0.000679231	1.66407E-66	8.94064E-24
**Striatum**	2.04186E-06	0.002388912	0.000679231		7.3896E-44	9.44197E-24
**Cerebellum**	3.96009E-38	5.94995E-64	1.66407E-66	7.3896E-44		3.83283E-23
**Olfactory bulb**	1.62504E-11	6.07646E-40	8.94064E-24	9.44197E-24	3.83283E-23	

Number Of Segments

Males	Cortex	Hippocampus	Amygdala	Striatum	Cerebellum	Olfactory bulb
**Cortex**		2.98806E-05	0.056774202	9.27465E-06	2.88901E-32	7.4098E-08
**Hippocampus**	2.98806E-05		0.002185278	0.377794456	2.40175E-35	1.4683E-15
**Amygdala**	0.056774202	0.002185278		0.000821102	3.7054E-42	5.2596E-12
**Striatum**	9.27465E-06	0.377794456	0.000821102		3.03084E-36	3.19565E-16
**Cerebellum**	2.88901E-32	2.40175E-35	3.7054E-42	3.03084E-36		2.45765E-10
**Olfactory bulb**	7.4098E-08	1.4683E-15	5.2596E-12	3.19565E-16	2.45765E-10	

Females	Cortex	Hippocampus	Amygdala	Striatum	Cerebellum	Olfactory bulb
**Cortex**		2.68645E-16	0.015563001	1.26903E-06	4.34358E-40	2.05102E-09
**Hippocampus**	2.68645E-16		1.93073E-12	0.000788858	6.72253E-66	2.24152E-38
**Amygdala**	0.015563001	1.93073E-12		0.000576543	1.79847E-69	2.70572E-20
**Striatum**	1.26903E-06	0.000788858	0.000576543		4.97708E-45	1.08184E-21
**Cerebellum**	4.34358E-40	6.72253E-66	1.79847E-69	4.97708E-45		1.04573E-28
**Olfactory bulb**	2.05102E-09	2.24152E-38	2.70572E-20	1.08184E-21	1.04573E-28	

Number Of Extremities

Males	Cortex	Hippocampus	Amygdala	Striatum	Cerebellum	Olfactory bulb
**Cortex**		0.000160761	0.227389948	0.000127268	3.18074E-30	9.33984E-11
**Hippocampus**	0.000160761		0.000915295	0.469146722	7.43509E-34	7.59506E-18
**Amygdala**	0.227389948	0.000915295		0.000733106	1.91109E-37	8.41574E-14
**Striatum**	0.000127268	0.469146722	0.000733106		1.86032E-34	4.85935E-18
**Cerebellum**	3.18074E-30	7.43509E-34	1.91109E-37	1.86032E-34		2.9345E-07
**Olfactory bulb**	9.33984E-11	7.59506E-18	8.41574E-14	4.85935E-18	2.9345E-07	
**Females**	**Cortex**	**Hippocampus**	**Amygdala**	**Striatum**	**Cerebellum**	**Olfactory bulb**
**Cortex**		3.59151E-16	0.013392231	1.17286E-06	1.60478E-41	1.05024E-10
**Hippocampus**	3.59151E-16		5.02274E-12	0.001000574	2.3543E-65	8.10194E-39
**Amygdala**	0.013392231	5.02274E-12		0.000683687	1.55482E-68	9.75674E-22
**Striatum**	1.17286E-06	0.001000574	0.000683687		3.93222E-45	1.5217E-22
**Cerebellum**	1.60478E-41	2.3543E-65	1.55482E-68	3.93222E-45		3.32942E-24
**Olfactory bulb**	1.05024E-10	8.10194E-39	9.75674E-22	1.5217E-22	3.32942E-24	

P>0.01, P<0.01

Total branches Length

Males	Cortex	Hippocampus	Amygdala	Striatum	Cerebellum	Olfactory bulb
**Cortex**		0.00053949	0.113545664	2.29673E-07	2.02418E-29	1.0602E-15
**Hippocampus**	0.00053949		0.008002707	0.049743963	3.23741E-31	7.43908E-21
**Amygdala**	0.113545664	0.008002707		1.01135E-05	6.89236E-38	4.22664E-21
**Striatum**	2.29673E-07	0.049743963	1.01135E-05		3.17421E-38	3.69385E-27
**Cerebellum**	2.02418E-29	3.23741E-31	6.89236E-38	3.17421E-38		0.000723871
**Olfactory bulb**	1.0602E-15	7.43908E-21	4.22664E-21	3.69385E-27	0.000723871	
**Females**	**Cortex**	**Hippocampus**	**Amygdala**	**Striatum**	**Cerebellum**	**Olfactory bulb**
**Cortex**		1.3232E-14	0.003613947	3.2344E-08	1.59067E-41	2.46128E-16
**Hippocampus**	1.3232E-14		6.44659E-09	0.033411594	8.3551E-65	9.53954E-44
**Amygdala**	0.003613947	6.44659E-09		0.000308527	6.59309E-65	1.11211E-30
**Striatum**	3.2344E-08	0.033411594	0.000308527		4.59964E-47	5.48827E-30
**Cerebellum**	1.59067E-41	8.3551E-65	6.59309E-65	4.59964E-47		5.84721E-16
**Olfactory bulb**	2.46128E-16	9.53954E-44	1.11211E-30	5.48827E-30	5.84721E-16	
